# Housing, Husbandry and Welfare of a “Classic” Fish Model, the Paradise Fish (*Macropodus opercularis*)

**DOI:** 10.3390/ani11030786

**Published:** 2021-03-11

**Authors:** Anita Rácz, Gábor Adorján, Erika Fodor, Boglárka Sellyei, Mohammed Tolba, Ádám Miklósi, Máté Varga

**Affiliations:** 1 Department of Genetics, ELTE Eötvös Loránd University, Pázmány Péter stny. 1C, 1117 Budapest, Hungary; foderi@gmail.com; 2 Budapest Zoo, Állatkerti krt. 6-12, H-1146 Budapest, Hungary; halbiologia@gmail.com; 3 Fish Pathology and Parasitology Team, Institute for Veterinary Medical Research, Centre for Agricultural Research, Hungária krt. 21, 1143 Budapest, Hungary; sellyei.boglarka@atk.hu; 4 Department of Zoology, Faculty of Science, Helwan University, Helwan 11795, Egypt; tolba_124@science.helwan.edu.eg; 5 Department of Ethology, ELTE Eötvös Loránd University, Pázmány Péter stny. 1C, 1117 Budapest, Hungary; adam.miklosi@ttk.elte.hu

**Keywords:** paradise fish, fish husbandry, fish welfare, fish housing, courtship behavior, anabantoid fish, labyrinth fish, bubble nest, Anabantidae, *Macropodus opercularis*

## Abstract

**Simple Summary:**

Paradise fish (*Macropodus opercularis*) has been a favored subject of behavioral research during the last decades of the 20th century. Lately, however, with a massively expanding genetic toolkit and a well annotated, fully sequenced genome, zebrafish (*Danio rerio*) became a central model of recent behavioral research. But, as the zebrafish behavioral repertoire is less complex than that of the paradise fish, the focus on zebrafish is a compromise. With the advent of novel methodologies, we think it is time to bring back paradise fish and develop it into a modern model of behavioral and evolutionary developmental biology (evo-devo) studies. The first step is to define the housing and husbandry conditions that can make a paradise fish a relevant and trustworthy model. Here, we define the relevant welfare parameters for keeping a healthy population of paradise fish and provide a detailed description of our recent experience in raising and successfully breeding this species under laboratory conditions.

**Abstract:**

Thanks to its small size, external fertilization and fecundity, over the past four decades, zebrafish (*Danio rerio*) has become the dominant fish model species in biological and biomedical research. Multiple lines of evidence, however, suggest that the reliance on only a handful of genetic model organisms is problematic, as their unique evolutionary histories makes them less than ideal to study biological questions unrelated to their historically contingent adaptations. Therefore, a need has emerged to develop novel model species, better suited for studying particular problems. The paradise fish (*Macropodus opercularis*) has a much more complex behavioral repertoire than zebrafish and has been a favored model animal in ethological research during the last decades of the previous century. We believe that with currently available, easily adaptable genetic toolkits, this species could be easily developed into a popular model of behavioral genetics. Despite its earlier popularity, however, the description of a detailed housing and husbandry protocol for this species is still missing from scientific literature. We present here a detailed description of how to raise and breed paradise fish successfully under laboratory conditions, and also discuss some of the challenges we faced while creating a stable breeding population for this species in our facility.

## 1. Introduction

Paradise fish, *Macropodus opercularis* (Linnaeus, 1758) is a small sized (8–11 cm long), obligatory air-breathing, tropical freshwater fish species. It is native to Southeast Asia [1,2,3,4,5], where its natural habitat is in small ponds, rice paddies and swamps covered with dense vegetation [1,4,6]. Floating plants are usually dominant in its spawning areas and they are necessary for their specialized nest-building activities [4]. 

The species belongs to the group of anabantoids (order Perciformes, suborder Anabantoidei), most of which are freshwater fish native in Africa and Asia [7,8,9]. There are roughly 137 anabantoid species worldwide that group into three families: Anabantidae, Helostomatidae and Osphronemidae, with paradise fish belonging to the latter [7,8]. 

The anabantoids are also known colloquially as “labyrinth fish” because they evolved a specialized organ that helps them to use the surface air for gas-exchange [4,7,8,9]. This labyrinth organ (LO), the special air-breathing apparatus, is housed in a pair of supra-branchial chambers. It is a bony structure made up of highly vascularized and folded membrane located next to the gill cavities [7,9]. Due to the presence of the LO, the gills of labyrinth fish are relatively small and their primary function is to excrete ammonia and CO_2_. Many anabantoid species are therefore obligatory air breathers and must surface to breath air to survive [8]. However, the presence of the LO also makes these fish well-adapted for some extreme environments. They can invade habitats where other fish species are not able to survive due to the extremely low levels of dissolved oxygen and/or slightly acidic pH ranges (such as hypoxic or polluted waters) [7,8]. While fish from this group can adapt well to extreme environmental conditions, current results suggest that paradise fish is a relatively fastidious species which does not tolerate extreme changes in the environmental conditions [10]. 

Air-breathing also influences a wide range of behaviors in the labyrinth fish, including territorial display, social communication, courtship, breeding and parental care [7,8]. These fish are best known for their unique reproductive behavior, which includes bubble nest-building, “anabantoid embrace” and parental care [5,7,11,12]. Depending on their egg types, some anabantoids produce floating eggs, others lay sinking eggs and some are sinking egg mouth brooders [9,13]. Paradise fish are floating egg producers and foam nest-builders. Their floating eggs are small (approximately 0.8–0.9 mm in diameter) and their yolk contains a large oil droplet. This makes the eggs lighter than water, so they are buoyant and rise to the surface once laid and fertilized. As the oil droplet is present until 3–4 days post-fertilization (dpf), the larvae are also buoyant and stay in the foam nest for the first few days after hatching [7,9]. 

Foam nest-builders can build their nests either on the surface of the water or under water (depends on the species) [9]. Surface foam nest-builders usually need floating plants or other substrates to hold the nest in place [4,8,9,14]. Bubble nest-building is performed by the male fish, who will also guard the nest until the larvae are old enough to start feeding on their own. The development of the nest-building behavior is strongly dependent on the presence of the LO as male fish gulp air on the surface and mix it with mucus secreted by the labyrinth organ to create the bubbles that will be attached to the surface vegetations [7,11,15]. 

This unique set of behaviors make the labyrinth fish ideally suited for a range of studies. Indeed, not so long ago, the paradise fish was a popular model for ethologists, and was studied for different aspects of its behavioral repertoire [3,5,6,16,17,18,19,20,21,22,23,24,25,26]. With the advent of novel molecular techniques, however, other, more established laboratory species (mainly zebrafish (*Danio rerio*) and medaka (*Oryzias latipes*)), replaced paradise fish in the realm of behavioral studies as well. 

*M. opercularis* was first introduced to Europe in 1869, where it became popular first in France, but soon it was available also in the UK and USA for hobby aquarists [3]. Interestingly, while the paradise fish has been a popular ornamental trade fish for over one and a half centuries, there is still relatively little known about its natural history. Similarly, despite its frequent use in ethological research in the latter decades of the 20th century, there are no detailed protocols for its captive housing and for the optimal husbandry techniques [2,3,4,5]. 

Besides being “good practice” that helps experiments, optimal husbandry should also take into account that new research guidelines aim to cause minimal discomfort to the studied animals [27,28]. The complexity of the paradise fish behavioral repertoire is similar to that of some birds or mammals, suggesting that fish are most likely sentient beings [27,28,29,30,31,32], which is also supported by the presence of analogous brain structures and functions to other vertebrates [33,34]. Multiple lines of evidence also support that fish (in general) possess more complex cognitive abilities than often given credit for [30]. As sentient beings fish are cognizant, can feel comfort and discomfort, therefore it is unethical to (purposely or through neglect) make them experience unnecessary discomfort or pain [34]. Protocols for handling and housing paradise fish should also reflect these concerns. 

As we are interested to develop *M. opercularis* into a modern model of behavioral studies, our primary goal is to establish the best husbandry and housing techniques for the species under scientific laboratory conditions. We present here a short assessment of our results and also discuss the challenges we encountered during this process, with the aim to present recommendations on how to start housing of this species in scientific laboratories. 

## 2. Materials and Methods 

### 2.1. Animals, Original Housing and Husbandry Conditions

Paradise fish used in this study were maintained and bred in the animal facility of the Institute of Biology at ELTE Eötvös Loránd University. All experimental procedures were approved by the Hungarian National Food Chain Safety Office (Permit Number: PE/EA/406—7/2020). 

The fish were imported into the facility from a local breeder at embryonic developmental stages (less than ~10 h old). The imported embryos were disinfected with a wash of KMNO_4_ (at 100 mg/L concentration for 2 min) followed by a NaClO (“bleach”) treatment (at 50 mg/L concentration for 5 min) and raised for 2–3 days in a 28.5 °C incubator. On the fourth day, the hatched and surviving larvae (180 in total) were transferred to the fish facility.

In the facility, larvae were kept in 3 L plastic containers at the density of 20–40 individuals per container for the first month. The containers had no automated filtration, therefore water quality was maintained with daily cleaning and manual water changes. As environmental enrichment, we added a few biofilter pebbles into each container, which also helped maintaining water quality. Room temperature was kept between 24 and 28 °C, and photoperiod was set on a 13 h Light:11 h Dark cycle. Fish water was buffered to 350 µS conductivity and pH 7.5.

Larvae were fed thrice a day with a mixture of commercial tropical and zebrafish food (NovoTom (JBL GmbH & Co. KG, Neuhofen, Germany, 2019), Sera Micron (Sera, Heinsberg, Germany, 2019), SDS 100, SDS 200, SDS 300 (Special Diet Services, Essex; UK 2019); Zebrafeed (Sparos Lda., Olhão, Portugal, 2019), TetraMin baby (Tetra GmbH, Melle; Germany 2019)). This diet has been supplemented with freshly hatched (24 h old) *Artemia salina* nauplii larvae as live food. 

Approximately 40% of the larvae (70/180) survived to the age of 1.5–2 months, when they were moved into glass aquaria with bio-sponge filtration. Forty individuals were placed into a ~25 L aquarium (57 × 20 × 27 cm), whereas the remaining 30 individuals were placed to a ~20 L aquarium (50 × 20 × 25 cm). We continued to use biofilter pebbles as enrichment. 

**Figure 1:**
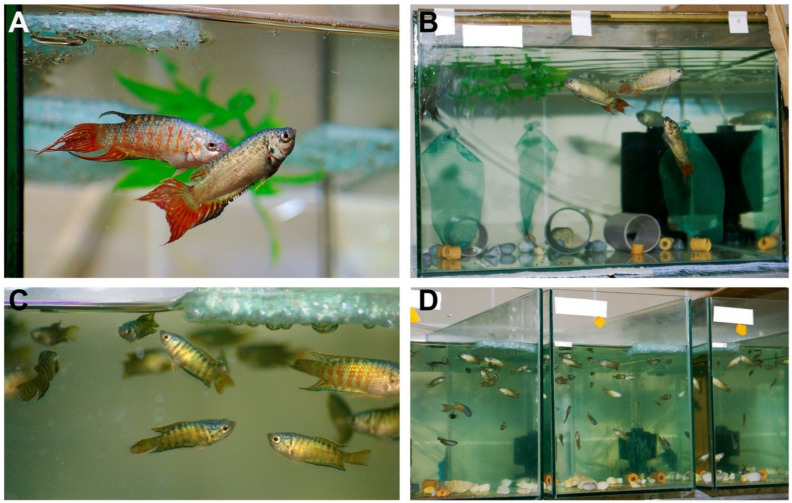
Housing conditions for paradise fish. (**A**) Adult, sexually dimorphic paradise fish pair. Female in the front, male in the back. (**B**) Adult paradise fish are housed at ~1 fish per 3 L water stocking densities, in covered, environmentally en-riched aquariums. (**C**) Adolescent (~4 months post-fertilization (mpf)) fish, with not fully developed sexual dimorphism, can be stocked at higher densities. (**D**) Housing conditions of juvenile paradise fish (~2 fish per 1 L of water). Photo credit: Anita Rácz.

When fish reached 3 months post-fertilization (mpf), we placed them into four larger glass aquariums (46 × 35 × 24 cm ~ 38.5 L) with approximately 31 L of actively used water volume in each. As only 65 individuals (derived from the imported embryos) survived to this age, the stocking density was about 15–17 individuals per aquarium. For additional environmental enrichment, we introduced small plastic floating plant leaves and green Styrofoam pieces (Figure 1A,B). 

Sexual dimorphism started to become apparent at ~5 mpf (adolescence) and was fully visible by 7–8 mpf (Figure 1C). At this stage, we stocked the fish at a final density of 10–12 individuals per aquarium and equal female-to-male ratios (same aquarium size as mentioned above for 3 mpf). To reduce the frequency of aggressive behaviors, we introduced further environmental enrichment: pieces of plastic pipes were placed into each tank to provide shelter areas for the fish (Figure 1B). It is important to emphasize that paradise fish tanks should be covered at all times. 

Water parameters were kept as above and regularly tested (temperature was read and recorded daily, pH and conductivity weekly—by computerized readers, while NH_3_, NO_2_^−^ and NO_3_^−^ levels were tested once a month by JBL test kits (JBL GmbH & Co. KG, Nenhofen; Germany 2019)). After 5 months, the feeding regime changed for twice daily feeding. To feed adult paradise fish, we used a mixture of commercial dry adult zebrafish and tropical fish food (SDS small granular food and Tetra Min flake food), supplemented with 48 h old, freshly hatched *Artemia* nauplii.

Environmental parameters are summarized in Table 1.

### 2.2. Breeding Conditions

While the natural spawning season of the species in its natural habitat occurs during the monsoon season (from May to October), with a peak between May and July [4], with appropriate environmental settings (light cycle and temperature), paradise fish can also breed out of their reproductive season under laboratory conditions [4]. 

Fish breeding in our facility started when the animals were older than 6 months. Breeding occurred continuously from November to the end of summer. Breeding couples were rotated weekly and set up in 15 L aquariums (40 × 25 × 25 cm). To help the males with their nest-building activities, we provided in each breeding tank five small plastic floating plant leaves, five pieces of green Styrofoam (for bubble nest-building) and two pieces of plastic pipes (shelter area for females). 

Fish were generally introduced to their breeding aquariums on Friday afternoons, and after an acclimatization period during the weekend, mating started at the beginning of the following week. Courtship and parental behaviors could be readily observed in these breeding pairs (Figure 1A).

### 2.3. Improved Housing Conditions

During the first year of housing, we encountered some diseased individuals in our breeding population and we also faced some difficulties with fish rearing. To improve housing conditions, we introduced a RO (re-osmosis water filter) unit (Ecosoft Robust 1000; Ecosoft Water Systems GmbH, Nettetal; Germany 2019) to our facility, and for our weekly water changes, we used water that was buffered to the desired parameters (350 µS, pH 7.5) from outer sump containers. To ensure the stability of pH in the holding tanks, we added sterilized coral crumbs and limestone pebbles into the bio-filter sponges and as substrate materials into the tanks.

We also improved the environmental enrichment in the holding aquaria by introducing vertically floating green mosquito net pieces to imitate water plants (so as to give structural area boundary and shelter) (Figure 1B). Coral crumbs, bio-pebbles and floating plant-like structures were also introduced into the rearing tanks of juvenile fish.

After successfully rearing the first generation of paradise fish in our facility and stabilizing breeding, we also started testing different rearing conditions.

In our first trials, we used either 10 L aquariums or 5 L plastic containers, both equipped with bio-sponge filter units. When using 10 L glass aquariums, we raised either 100 or 50 individuals in each of the 3 aquariums used in parallel. We performed this trial twice, using a total of 450 larvae. In case of the 5 L plastic containers, the trial was repeated 3 times, and for every trial, we placed 50–70 individuals in each of the 2 containers used in parallel. A total of 360 larvae have been tested under this condition.

When we abandoned sponge filtration units and switched back to using standing water, we started using 3 L plastic containers. Using these conditions, different initial rearing densities have been tested in parallel holding designs. We compared the efficiencies of rearing 100 individuals per 3 L container (a total of 400 individuals tested), 70 larvae per 3 L container (a total of 350 larvae tested) and 50 individuals per 3 L container (a total of 350 larvae have been tested).

When testing for different diets, larvae were either fed exclusively with powdered dry food until the end of the third week, when we started to give them freshly hatched *Artemia* nauplii as well, or they were given a mixture of nauplii and dry food immediately from 6 dpf.

For all tested groups and conditions, individuals that reached the age of 12–13 wpf survived to adulthood.

### 2.4. Behavioral Observations

Observations of the group behaviors (social interaction and territorial defense) were carried out in the holding aquariums before and after fish have been moved into and back from breeding tanks. Nest-building and courtship behavior could be observed in the breeding aquariums (see above). For a complete list of paradise fish behavioral elements see Table A1.

### 2.5. Health Diagnostics

Diseased paradise fish were tested with both classical, bacteriological and pathological methods, and molecular (PCR) diagnostics. 

Bacteriology and pathological examinations were carried out by the Fish Pathology and Parasitology Team at the Institute for Veterinary Medical Research (IVMR) of the Centre for Agricultural Research (CAR). The autopsy included visual and microscopical observations of the fish after euthanasia. For bacteriological study, swab samples were collected from wounds and streaked on 10% sheep blood agar, Aeromonas agar, MacConkey agar and tryptic soy agar plates. In addition, direct isolation was performed from main inner organs (including gill, labyrinth organ, liver, spleen and kidney) on the same media. The plates were incubated for 24–48 h at room temperature (average 20–22 °C). The colonies growing on agar plates were characterized by their appearances, visually and by light microscope, and identified taxonomically by molecular methods. In the preliminary examinations, the morphology and pigment production of the bacterial colonies were observed. In native smears, the shape and movement of each bacteria were checked by microscopy. Finally, selected unique bacterial colonies were subjected to universal 16S rDNA PCR and sequencing for taxonomical classification [35].

Bacterial 16S rDNA was amplified from colonies by using the following primers: forward primer 5′-AGAGTTTGATCMTGGCTCAG-3′ and reverse primer 5′-GGTTACCTTGTTACGACTT-3′). The 25 µL PCR reactions were assembled using DreamTaq reagents (Thermo Fisher Scientific Inc. Waltham, MA, USA), primers and template DNA. The PCR amplification program included 4 min of initial denaturation at 94 °C, then 35 cycles at 94 °C for 45 s, annealing at 55 °C for 60 s and at 72 °C for 45 s, and then 3 min of the final elongation at 72 °C, and was executed in a 2720 Thermal Cycler (Applied Biosystems, Foster City, CA, USA). The approximately 1400 bp large fragments were purified for direct sequencing using the Geneaid PCR purification Kit (Geneaid Biotech, Ltd., Taipei, Taiwan). Fragments were sequenced in both directions with the PCR primers using the BigDye Terminator Ready Reaction Mix v3.1. Cycle Sequencing Kit (Perkin-Elmer, Applied Biosystems, Foster City, CA, USA) according to the producer’s recommendations, and sequencing reactions were run on an ABI Prism 3100 Genetic Analyzer (Applied Biosystems, Foster City, CA, USA). Taxonomic identification homology searches were carried out in GenBank by using the Basic Local Alignment Search Tool (BLAST) algorithms on the National Center for Biotechnology Information (NCBI) server.

Molecular diagnostics was carried out with the Circulum Sampling Kit diagnostics package provided by QM Diagnostics (Nijmegen, The Netherlands). Following the recommendations of two earlier publications, describing health monitoring techniques in zebrafish facilities, we used the recommended environmental screening approaches for our sample collection [36,37]. Sample processing and analysis was carried out by QM Diagnostics, following the company’s in-house protocols. The analyzed specimens included a symptomatic dead fish sample, a mixed biofilm sample from one of the main holding aquariums, a mixed sludge sample from symptomatic individuals and a mixed sludge sample from the main housing tanks. The applied Circulum Sampling Kit PCR panel consisted of primers specific for *Aeromonas hydrophila*, *Flavobacterium columnare*, *Mycobacterium* spp., *Pseudocapillaria tomentosa*, *Pseudoloma neurophilia* and *Pseudomonas aeruginosa*. In the case of *Mycobacterium* spp. positivity, an additional mycobacteria-specific panel for the 6 most common aquarium species (*M. abscessus*, *M. chelonae*, *M. fortuitum*, *M. haemophilum*, *M. marinum* and *M. peregrinum*) was also used. The Circulum Kit sampling profile consists of high sensitivity, highly specificity and fast TaqMan qPCR and chemical assays, which are all validated by QM Diagnostics Company standards. In the sample preparation process, the company homogenizes the whole body of a fish in sterile phosphate-buffered saline buffer (PBS) and uses this sample for total nucleic acid purification. Swabs were vortexed in 1.5 mL tubes containing solution and then discarded with 175 μL of the sample supernatant being used for the DNA extraction. Sludge was diluted in sterile saline solution and the mixture was used for total nucleic acid purification.

### 2.6. Anesthesia and Analgesia 

For certain body measurements or for some genetic/molecular experiments where tissue collection is needed (for example fin-clipping), the fish need to be anesthetized for shorter or longer periods of time. To perform these experiments, we adapted zebrafish fin-clipping protocols [38] for paradise fish. Our optimized protocol combines a safe dosage of the anesthetic agent Tricaine (MS-222) (final concentration: 120 mg/L) with the use of an analgesic agent, Lidocaine (final concentration: 4–5 mg/L), to reduce potentially painful stimuli under the small surgical process [38,39,40,41] (for the detailed protocol, see the Appendix B).

### 2.7. Data Visualization

Data visualization was performed in R [42] using the *ggplot2* package [43]. All figures have been assembled in Affinity Designer v. 1.7.3. (Serif Europe).

## 3. Results

### 3.1. Environmental Factors and Optimal Housing Conditions

We could not find any data in the literature about the survival rate of paradise fish embryos in their natural habitat, but with our initial conditions (rearing paradise fish larvae in 2–3 L plastic containers with daily cleaning and manual water changes), approximately 35% (65/180) of the fertilized eggs reached adulthood.

Further on, using the offspring of our founder population, we tested alternative conditions and observed that different stocking densities, feeding regimes and the type and size of the rearing tanks all affected survival rates (Table 2). We started by comparing the survival rates of larvae reared in different water volumes and stocking densities. First, we housed the larvae in 10 L glass aquariums with biofilter-sponge filtration systems. Using a stocking density of either 50 or 100 individuals per 10 L aquarium, however, resulted in the loss of all larvae within 1–2 weeks in the repeated trials. Next, we tested 5 L plastic containers with sponge filtration. In these trials, 50–70 individuals were placed in each container. Again, unexpectedly, all the larvae reared were lost within 2–4 weeks. 

Once we abandoned sponge filtration and started to rear paradise fish larvae at low larval stocking densities, in standing water bodies with smaller water volume and daily water changes, survival increased. Different rearing densities in 3 L plastic containers have been tested in parallel holding designs for 100, 70, 50 and 20–40 larvae per container. The results of all these trials are summarized in Table 2.

Several studies found in other anabantoid species that a mixed diet feeding (the inclusion of live food) into the diet of larval fish increased the growth rate and survival rate of the fish [14,44,45,46]. Supplementation of the diet with live food (freshly hatched 24 h old *Artemia* nauplii) from 6 dpf also helped us to increase the survival rate of larvae compared to batches where *Artemia* has just been added after 3 weeks post-fertilization (wpf) into the diet. After the first month, 48 h old *Artemia* nauplii could also be used for feeding. With these rearing conditions, we managed to raise 20–40% of fertilized embryos to adulthood (Table 2).

**Figure 2:**
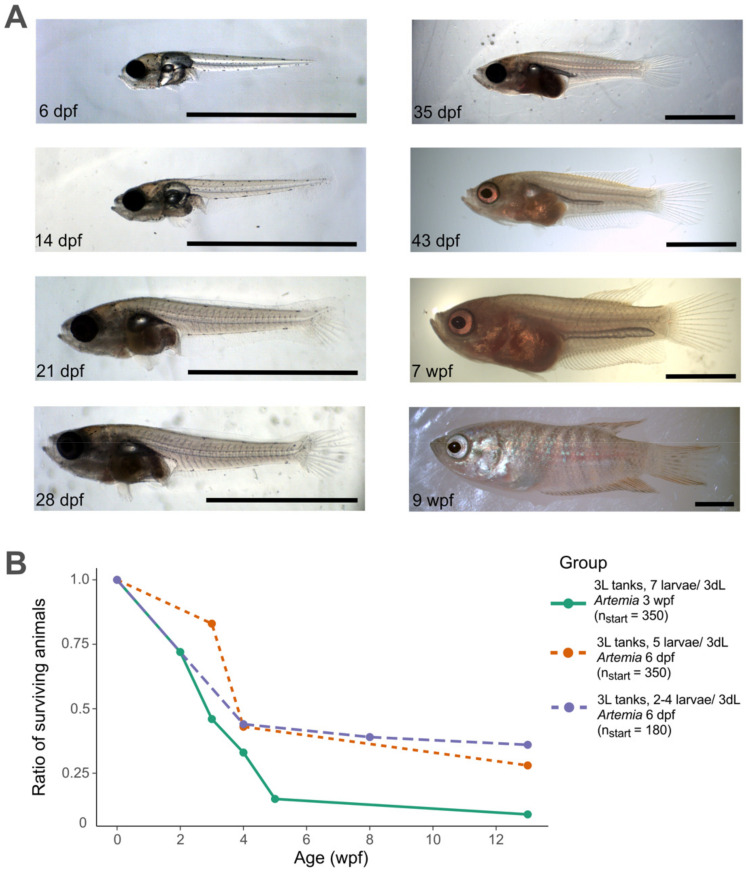
Larval/juvenile development of *M. opercularis*. (**A**) A larval/juvenile developmental series of paradise fish. Scale bars are 1 mm. (**B**) Larval survival rate under different conditions tested. (dpf = day of post-fertilization, wpf = week of post-fertilization, n_start_ = number of larvae at the beginning of the observation period). Photo credit: Máté Varga.

Like other teleost, paradise fish undergo a period of metamorphosis while transitioning from larval to juvenile stages [12,47]. For many fish species, this is a critical period with high mortality rates [48,49]. In our facility, we also observed high larval mortality between 3 and 5 wpf that coincided with the period of morphological change for paradise fish species [12] (Figure 2). While up to 50–60% of larval fish died in this two-week period, before and after metamorphosis, mortality was minimal (Figure 2B).

After 6–7 wpf, juveniles were relocated into 10–15 L glass aquariums equipped with pumps and bio-sponge filters at the density of ~2 juveniles per 1 L of water. After an additional 6–8 weeks, young adults were relocated again (to aquariums with 31 L active capacity) to achieve lower stocking densities of ~1 fish per 2 L of water. Lower stocking densities were necessary for faster growth and lower aggression. Juvenile sibling fish were not aggressive with each other so they could be housed in larger groups. Once they reached sexual maturity and sexual dimorphism became apparent, however, fish displayed more aggressive behavior. We observed male–male, female–female and male–female fights, the number of which was considerably reduced when we decreased the stocking density. 

After 5–6 mpf, fish were kept in stable groups in their holding tanks, where the formation of hierarchies could be observed (final density of ~1 fish per 3 L in glass aquariums with 31 L active capacity). Additional environmental enrichment elements and sheltering structures were introduced into the holding aquariums to help the fish to avoid aggressive individuals (Figure 1B). Using the aforementioned housing conditions, we successfully raised ~200 paradise fish and we were able to maintain a stable population of ~160 individuals in our facility.

Our initial results suggested that water parameters and feeding regime optimized for zebrafish (Table 1) were optimal for paradise fish husbandry as well (pH range 7–8, conductivity range 300–600 µS, temperature 24–28 °C, 13 h Light:11 h Dark cycle) [49,50,51]. Later, however, some of our fish started to show different symptoms of disease (sores on the body surface or on/around the mouth, redness and inflammation of the anal/cloacal site/region in males). Searching for a potential cause, we realized that despite regular, weekly water changes, the pH level fluctuated significantly in the holding tanks. After 4–5 days following each water change, the sodium-bicarbonate buffering capacity was depleted and rapid acidification occurred. The pH in the holding tank fluctuated, therefore, between ~7.5 to ~5.8 on a weekly basis, the latter being outside of the optimum range. We were able to improve holding conditions with the introduction of coral crumbs and limestone pebbles into the biofilter sponges and in the main tank areas. Both coral crumbs and limestone pebbles start to dissolve in acidic environments and with their use, we were able to stabilize the pH in the tanks, keeping it constantly in the pH 7.0–7.6 range.

We also added frequently (monthly–bimonthly) nitrifying bacterial cultures to our tanks. These kept the biological filters stable in the aquariums, and we did not detect any harmful levels of NH_3_, NO_2_^−^ and NO_3_^−^.

### 3.2. Social Behavior and Courtship

After reaching adolescence (4–5 mpf), paradise fish started to develop their sexually dimorphic traits. Males elongated their dorsal, anal and caudal fins, while females developed rounder bellies. Coloration differences also became apparent between the sexes. Males displayed bright colors in a wide range of combinations (red or blue), depending on their behavior, while females usually lost some of their coloration to become pale-silvery. 

In parallel with the appearance of these sexually dimorphic traits (Figure 1A), fish also became more aggressive after 5 mpf. Ranking fights occurred in the tank and led to the emergence of a hierarchical structure. Once this was achieved, the highest-ranking male started to dominate the frontal area of the tank (where food was coming in) while subordinate males resided in the middle and back areas of the tanks. 

These settled hierarchies, however, could be easily perturbed. Removing individual fish for short breeding sessions and later (after ~5 days) returning them to the holding tanks frequently resulted in the resumption of aggressive behavior and intense fights (Supplementary Video S1).

#### 3.2.1. Species-Specific Behavioral Elements

Paradise fish are well-known for their ritualized display behaviors [5,16,17,18] that reflect an individual’s physiological condition (e.g., breeding, social status or territoriality) and are also important for inter-group communication [7]. 

We provide here a concise list and short description of the most common behaviors observed in our facility. Where possible, we try to break these behaviors down to their elements as previously described in the paradise fish ethogram. For a more complete list and more precise description of the behavioral elements, see Table A1. We highlighted these behaviors as they can provide direct information about housing (i.e., if individuals are stressed either due to overcrowding or due to the lack of environmental enrichment elements, significantly more aggressive behavior can be observed, and this requires immediate action).

##### Frontal Spread Display and Opercular Erection (OPE)

It is a stereotypical, dominancy-related interaction which is highly costly for the individuals. Fish rotating their gill covers (opercula) generate hypoxic conditions in their body [7]. In full display, the median fins are erected usually perpendicularly towards the other individual, while the opercula are extended and rotated out (Supplementary Video S2). Opercular spots (ocelli) present on the side of opercula become highly visible for the opponent. Frontal display can extend into poking, biting, tail-beating, chasing and other aggressive behaviors [5,18]. 

##### Lateral Spread Display (LSD) and Tail Beating (TAB) 

**Figure 3:**
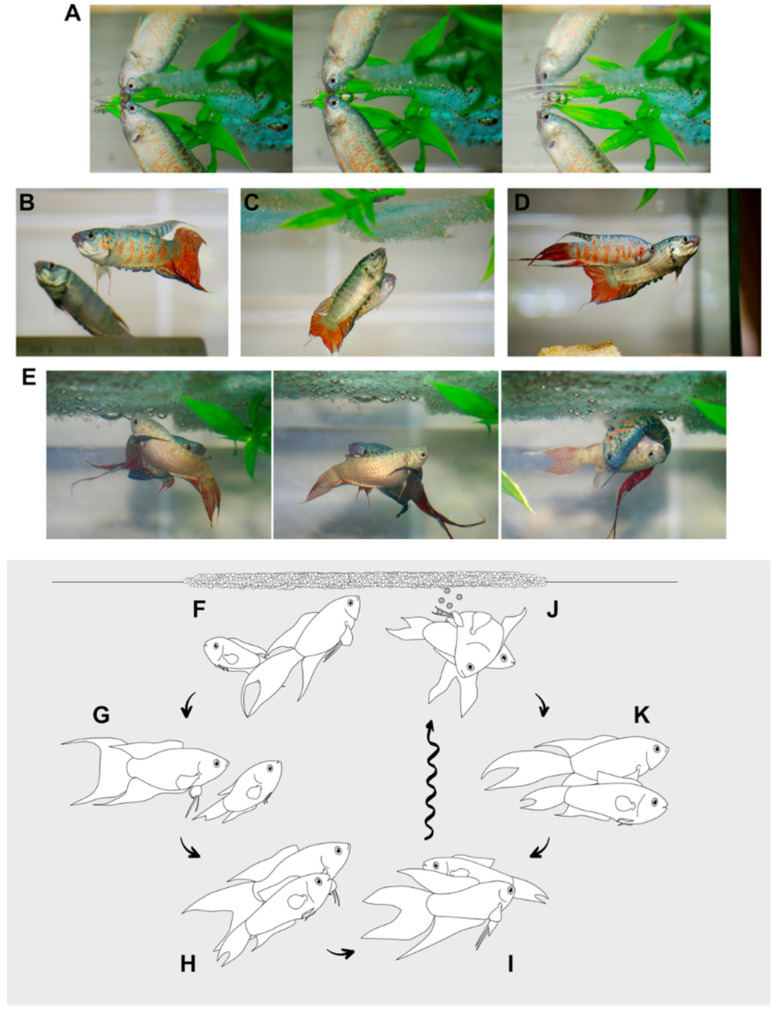
Reproductive behavior of the paradise fish. (**A**) Male paradise fish building nest, by ejecting mucus covered bubbles (**A**,**B**). (**B**) Paradise fish males show lateral spread display (LSD) behavior towards female fish in courtship. (**C**,**D**) Paradise fish courtship behavior: female fish check nest (**C**) or refuse male’s invitation by showing aggressive displays (OPE—opercular erection, LSD), vibrating movement (VIB) towards male fish (**D**), or even escape (ESC). (**E**) Paradise fish performing circling (CIR) followed by anabantoind embrace (ANE). (**F**–**K**) Step by step illustration of courtship behavior at *M. opercularis*. (**F**) Male fish building nest (**A**,**B**), (**G**) male fish starts lateral spread display (LHH), (**H**) male leads female to the bubble nest (LEN), (**I**) female shows a vertical waggle (VEW) followed by circle swimming (CIRC) under and toward nest, (**J**) anabantoid embrace (ANE) and release of gametes, (**K**) swimming inhibition (SIN). Steps I to K are repeated approximately 12–15 times, until the spawning process is finished (2–3 h). Drawing credit: Renáta Hamar, photo credit: Anita Rácz.

These behaviors are common during courtship when they are usually performed simultaneously (Figure 3B). During lateral display, a fish spreads its caudal and median fins in the front of or in parallel to the other individual. The tail beating is performed consequently by the displaying fish. In aggressive encounters, we may differentiate display at distance (DIS), head-head display (HHD) and head-tail display (HTD) [18] between individuals (Supplementary Video S2).

##### Biting (BIT)

One fish bites another one. Biting is associated with highly aggressive situations (between males or between a female and an aggressive male).

##### Chasing

The dominant fish charges (CHA) and the fleeing individual escapes (ESC) with folded fins (these signal submission, see below). Chasing can be short or prolonged, ending when the fleeing individual finds refuge, or the submissive display stops the aggression (CHA and or BIT) of the dominant fish.

##### Oblique Movements (OBM) and Vertical Waggle (VEW)

This posture is performed by females toward males or by submissive individuals toward dominant ones. The fins are folded, and the body may tilt vertically or horizontally. During mating, the lateral or vertical tilt usually changes into low-frequency tail beating vertical waggle (VEW) [5]. As an appeasement posture, this reduces aggressiveness and harassment.

##### Attack and Mouth-Lock (MOU)

When threatening displays are not enough to establish a winner, physical fight ensues. During mouth-lock, two individuals bite and lock their mouths/jaws together, while waggling with their body (Supplementary Video S1). This can last from a few seconds up to several minutes, in extreme situations. As time advances, body coloration becomes darker, probably due to the hypoxic conditions. While typically mouth-lock (MOU) is observed between males, occasionally we have seen it between females as well. Individuals in better physical condition can hold this position longer without the need to surface for breathing air. For similarly fit males, intense fighting could last up to ~20 min, until one participant gets hurt and surrenders [16,19]. Sometimes losers show oblique plan floating position (OBF) [16] or hide and rest (RES) after the lost fight. Fighting between males also attracts the attention of females who gather around and watch the combat (Supplementary Video S2).

#### 3.2.2. Reproductive and Courtship Behaviors

For the majority of developmental experiments, it is important to collect the eggs right after fertilization. Due to the prolonged courtship, however, in paradise fish, it can be challenging to predict when this will happen. Furthermore, disturbing the fish or the nest too early can not only cause unnecessary stress to the animals, but it can also interfere with the breeding process. Therefore, we provide a concise description of the behavioral and physiological changes that can be observed during breeding and can used to foresee spawning events. 

During courtship, males perform a combination of the behavioral elements used for territorial defense and social display (Figure 3F–K). Opercular erection, fin spreading, lateral display, poking and chasing all appear during courtship behavior, with bubble nest-building is a new, additional behavioral element (Table 3). A detailed, quantitative analysis for paradise fish courtship–spawning has been provided by Hall [5], whereas here we just provide a brief summary of these behaviors (for a list of behavioral unit abbreviations see Table A1).

Changes in body color to a brighter complexion are usually the first sign that indicates the readiness of spawning in males. At the same time, males start to chase the females, occasionally performing opercular raise (OPE) or lateral spread display (lateral head-head display–LHH or lateral head-tail display–LHT) in front of them. They also start to build nests by gulping bubbles of air into their mouth and eject them with a mucous coat under objects floating on the water surface (air-bubbling (A–B)) (Figure 3A). Females generally exhibit specific postures (e.g., OBM and VEW) if they are willing to accept the courting; otherwise, they either become aggressive towards the males (OPE, LSD, VIB—lateral vibrating movement) or (more frequently) flee (ESC) and hide from them.

Once the nest is ready, males will increase courtship towards receptive females. After performing lateral spread displays (LHH or LHT) (Figure 3B), they turn around and show the way to the nest by slowly swimming back to it (LEN—leads female to nest). If females do not follow, the sequence is repeated over. Females at this point either follow and check the nest (Figure 3C) or refuse the male either by escaping (ESC) or showing aggressive postures with opercular erection (OPE), display (DIP) and vibrating (VIB) (Figure 3D). Females ready for mating will perform a vertical waggle (VEW) and show their dorsal side to males as part of a slow oblique movement (OBM). Finally, the male and the female will swim under the nest where they circle around and start the spawning process (Figure 3E).

During spawning, the male curves its body, to touch its tail, with the head at mid-section of the female while turning females upside-down in a close embrace—this posture is called anabantoid embrace (ANE) [7,11] (Figure 3J). At this point, both fish start to tremble, release the eggs and milk and undergo a period of swimming inhibition (SIN) while they slowly sink to the bottom. 

Fertilized and unfertilized eggs will float upwards into the bubble nest. Some eggs might miss the nest, but males will pick them up later by mouth (PIC) and relocate them into the bubble nest. Occasionally, females also try to help, but at this point, males can become aggressive and chase them away, until egg collection is finished. Individual spawning actions can be separated by several minutes. Males and females repeat these embracing circles in frequencies with sometimes not just spawning but pseudo-spawning actions as well.

The whole mating process can last several hours (2–3 h on average) and by the end, the female releases approximately 400–600 eggs (usually 15–60 eggs per embrace). These results are in line with the observations of Ward [3]. In our facility, spawning usually started in the early morning hours, but never lasted longer than mid-day. 

Post-spawning males stay under the bubble nest and defend the territory around it vigorously. A set of nest-caring activities also ensues. Males continue to add new mucus-covered bubbles to the nest (A–B) and retrieve the embryos that escape from it (PIC). These activities continue until the hatched embryos are old enough to leave the bubble nest (~6 dpf).

### 3.3. Effects of Adverse Conditions on Paradise Fish Welfare 

#### 3.3.1. Incidence of Stress-Related Disease Symptoms

First signs of disease within our breeding colony appeared at 8 mpf (2 months after regular breeding has started). Affected individuals showed a variety of symptoms: scratches and open wounds on the body surface and inflammation around the mouth or cloacal area. Diseased individuals randomly appeared in different holding tanks, even after we applied strict quarantine for all the tanks, suggesting that we had to deal with an opportunistic pathogen and not an obligatory pathogenic microorganism.

**Figure 4:**
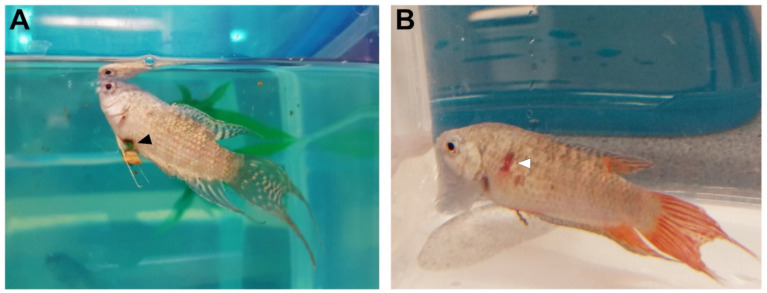
Disease symptoms of captive paradise fish. (**A**) Sick paradise fish male. Note the loss of color and the necrotized tissue (black arrowhead) in the region of the anal papilla. (**B**) Female with inflamed scratches on the body surface (white arrowhead). Photo credit: Tamás Annus and Anita Rácz.

Interestingly, some symptoms were strictly sex-specific. For example, only males showed cloacal inflammation, initially the region around the anal papilla got red and sore, followed by a slow necrotization, resulting in the formation of a hole in the body wall (Figure 4A). After the necrotized tissue detached, the cloacal region started to slowly regenerate. Affected males continued to eat and defecate during the whole period, however, with time, they constantly lost weight and became too weak after a few weeks. 

The other symptoms, red and inflamed mouth and inflamed scratches on the body surface, were typical for females (Figure 4B). Occasionally, they also became dropsy before dying in the quarantine tanks. 

After a period of investigation, we noted that only individuals used for breeding in the previous weeks tended to become sick. The incidence rate was higher among males than females. 

Our exploratory investigation also revealed that the pH of the holding tanks was fluctuating (see Section 3.1). While fixing the environmental factors, the prevalence of illness decreased, though diseased individuals still appeared occasionally, after being used in breeding sessions. We performed pathological and microbiological examinations, and in addition sent infected samples to QM Diagnostics to test them with a PCR panel for common, fish-specific pathogens. A summary of health diagnostics can be found in Table 4.

##### Results for Pathology and Bacteriology

During the autopsy, no pathological changes or traces of internal or external parasite infection associated with the lesions were found. In the microbiological examination, a low number of slightly diverse bacteria could be isolated. Among them, next to *Pseudomonas*, *Aerococcus* and *Comamonas*, *Aeromonas* species were dominant.

##### Results for Molecular Diagnostics (PCR)

The Circulum sampling Kit service provided by QM Diagnostics company tests for the presence of the following agents: *Aeromonas hydrophila*, *Flavobacterium columnare*, *Mycobacterium* spp., *Pseudocapillaria tomentosa*, *Pseudoloma neurophilia* and *Pseudomonas aeruginosa*. Using their PCR-based detection protocols, they could not detect any of the tested microorganisms in the mixed sludge sample from the holding tanks. The mixed sludge sample of the sick fish was positive for *Aeromonas hydrophila* and the mixed biofilm sample of the holding tanks for *Mycobacterium* spp. Moreover, the sick dead fish sample came back positive for *Mycobacterium* spp. and *Aeromonas hydrophila* as well.

The *Mycobacterium* spp.—positive samples were further tested by QM Diagnostics with their mycobacteria-specific PCR panel. This panel includes the six most common aquarium species (*M. abscessus*, *M. chelonae*, *M. fortuitum*, *M. haemophilum*, *M. marinum* and *M. peregrinum*). Although the biofilm sample got positive results with *M. chelonae* and *M. fortuitum*, in the specimen from sick dead fish, none of the 6 tested species could be detected, suggesting the presence of any additional mycobacterial species in the sick individuals and in the holding tanks.

#### 3.3.2. Anesthesia and Analgesia

Standard fish work required the adaption of anesthesia and fin-clipping protocols for paradise fish. In our trials, Tricaine at 120 mg/L concentration provided a safe and effective dose: all individuals became anesthetized within 3–5 min, depending on their body weight.

We noticed that the pigmentation of both males and females became darker as they lost control during the anesthesia, suggesting an active, continuous control of melanophore morphology. Interestingly, this was different from the change in coloration under stress, when the fish tended to become pale. 

After the performing of desired procedures (e.g., fin-clipping or body weighting), the anesthetized individuals were immediately placed into recovery tanks to avoid desiccation. Fish recovered fully within 3–5 min and they swam to the surface to gasp air several times. They also toned down their bright body coloration and after 4–5 min they started to behave normally.

All our fish (~15 individuals) subjected to anesthesia survived without suffering any lasting harm.

## 4. Discussion

Over the past decades, the enhanced/intensive application in the research of zebrafish and to a lesser extent medaka as dominant genetic model organisms has established fish as useful tools to study the etiology of human diseases and to search for novel drug precursor molecules [52,53,54,55]. Despite its numerous advantages, the increased number of evidence suggests that zebrafish as a model has its limitations and we should also use alternative fish species, that might be better suited for specific biological questions [56,57]. 

Thanks to its complex behavioral repertoire, in the 1970s, paradise fish emerged as a leading model for behavioral research [3,5,17,21,25,26,58,59,60]. In spite of some intriguing early results, however, thanks to the dynamic expansion of the genetic toolkit, zebrafish soon displaced paradise fish as well as the leading fish model for behavioral research. Yet, its more complex behavior still makes paradise fish an alluring model. Now, with the arrival of next-generation sequencing and novel genome editing and transgenesis techniques, we could aim to develop paradise fish into a leading model of behavioral genetics [61]. The aim of our work was to make the first step into this direction and rigorously define the housing and husbandry conditions for paradise fish, as—while the species has been used for behavioral research for decades—these conditions were never really specified [2,3,4,5]. 

Fish are poikilothermic aquatic animals who inhabit a complex three-dimensional environment which directly influences their welfare [62]. Water conditions outside of the species-specific optimal range can have long-lasting effects on fish physiology and welfare [29,34,63,64] as water quality is critical for ensuring the health and longevity of the fish [8]. Therefore, our first task was to define the optimal water quality and maintenance parameters for paradise fish [29,62]. 

In order to ensure optimal conditions, aquarium water should be continuously filtered or, if that is not an available option, should be changed on a regular basis [29]. A filtration system reduces manual care and can physically remove solid waste. It also provides a surface for the attachment of bacteria essential for the biological degradation of waste products (such as NH_3_) [29]. 

Our initial routine included weekly changes of the water in the holding tanks from a buffered water supply (pH 7.5, 350 µS). We soon realized, however, that after 3–4 days, a rapid acidification of the water occurred, and pH could drop as low as 5.8 by the end of the week. This occurred because biological filtration (and related bacterial activity) combined with the accumulation of waste products excreted by the fish, which gradually induce the acidification of the water [65]. This could be prevented by adding alkali agents, such as CaCO_3_—containing shells and corals, directly into the filter medium [65]. As these start to dissolve when the pH gets acidic, this simple, yet reliable intervention helped us to keep the pH levels in an optimal range (pH 7.0–7.6) at all times.

In tropical regions, where paradise fish are endemic, temperature and the length of the days are the main reproductive stimuli for fish and the normal breeding season lasts from May to October [4]. While occasionally our breeding pairs started spawning at temperatures as low as 23–24 °C, the optimal temperature range for bubble nest-building and breeding was 26–28 °C [4,9,11,12]. The light cycle optimized for the zebrafish facility (13 h Light:11 h Dark) also worked well for paradise fish. Using these conditions, our paradise fish colony bred continuously from November to the end of summer.

We also made an effort to provide sufficient environmental enrichment in our holding tanks. Unfortunately, as research laboratory set-ups are generally designed to standardize conditions, they are too often stimulus-poor, barren tanks/aquaria [62,66]. Several lines of research, however, show that a combination of structural, social and dietary enrichments can be beneficial for laboratory animals [28,62]. A structurally enriched, complex environment could provide animals hiding places and a sense of having control over their environment [28,29], which could reduce stress even in aversive situations [67]. Dietary enrichment, such as giving our fish live food, provides mental stimulation to capture prey and the opportunity for foraging, which promote a wider range of behavior [29,62]. Finally, social enrichment (keeping the animals in groups within the tanks) allows interaction between individuals, which is natural stimulus to them. However, finding the optimal stocking density can be challenging. A high stocking density leads to crowdedness, decreased water quality, stress and finally, a higher rate of disease. Too low stocking densities might lead to an unbalanced group structure with high levels of territorial aggression between individuals [28,62].

As paradise fish get older and larger, lower stocking densities are required for ideal growth and reduced aggression. Thus, while for juveniles 30–40 fish per 20 L of water was a usable stocking density, for adults, we had to decrease stocking densities to approximately 10–12 fish per 30 L. With the addition of extra structural elements to the tank (pieces of plastic pipes, floating plant leaves and vertically standing green mosquito net pieces), the group quickly established a hierarchy and fish lived together without excessive stress. 

Typical nest-builder fish, such as the paradise fish, also need floating plant-like materials to create their nests [4,8,9,14]. Therefore, in our breeding tanks, we provided green floating Styrofoam pieces which were indeed used by the males during their nest-building activities.

The rearing of anabantoid species has been shown to be a challenging task [14,15], and paradise fish are no exception [9]. With our current protocols, we are able to raise ~20–30% of the embryos to adulthood, so there is clearly space for improvement. Our results show that live feed (*Artemia* nauplii larvae) is essential for the larvae, as in their absence they grow slower and die at higher rates. Other studies came to similar conclusions for other anabantoids [14,29,45,46]. Further investigation is needed on the field of nutritional needs and feeding design for paradise fish to provide them a well-established diet for their specific requirements.

It is likely that including additional nutritional supplements, such as carotenoids, in their diet, could further improve the wellbeing of paradise fish stocks. Carotenoids for example can be important for pigment synthesis, but they also serve as a source of antioxidants and can modulate the immune response. Vertebrates are not able to synthetize carotenoids, so they have to uptake them through their food [68]. In the closely related bettas (*Betta splendens*), for example, the inclusion of carotenoids in the diet improved both the body colors and the immune response as well [29,68]. 

Once we established our housing and husbandry routines, we were also able to regularly observe some species-specific behavioral patterns. The development of the LO (as a ‘morpho-physiological innovation’) not only helped anabantoids to adapt to extreme environmental conditions, but also resulted in the evolution of unique physiological and behavioral features [7]. They also evolved behaviors that may inform other fish about the fitness of the performing individual. The rotation of the opercula, for example, is a very costly and unusual behavior for the fish, as it results in a self-imposed, energetically costly, hypoxic state. For the same reason, however, it is also a useful indicator about the physical condition of a male, as sick or weak individuals cannot afford to perform it at high frequencies [7]. 

Body color could also provide information about the physiological condition. Paradise fish can dynamically change their body coloration and adapt it to their behavior. These changes are more profound in males, especially during courtship (using intense red coloration), which might also provide an explanation as to why males often get sick post-mating: if carotenoid reserves in the body are depleted during breeding, fish might be prone for diseases as a robust immune response might also necessitate carotenoids, just as in closely related bettas (trade-off between red coloration and immune response) [29,68]. We hypothesize that just like in other species, there is a constant trade-off for paradise fish males between staying healthy, winning fights/protecting dominance and attracting mates. As most of the rituals related to breeding are energetically costly and/or demand high levels of O_2_ consumption, courtship and spawning action could be physiologically costly for paradise fish males (our latest observation suggests that when males were housed alone for an extra two days post-breeding, the number of hierarchical fights decreased when they were returned to their original tanks).

Finally, our observations also provided some insights into the emergence of disease in paradise fish. The random appearance of symptoms in unrelated individuals in different tanks suggested that an opportunistic pathogen/s could be behind the diseases. As immune suppression is known to lower the resistance to disease [63,64,69], we suspected that a transient immune suppression (as outlined above) induced by stress could be associated with the manifestation of disease, as affected individuals were always used for breeding in the previous weeks. Besides the energetically costly courtship and spawning behaviors, individuals were removed from their holding tanks before breeding. This disturbed the established hierarchy and the later reintroduction of the fish into their tanks of origin resulted in fights. In addition, the confiscation of fertilized eggs from the males protecting their nests was probably also stressful. All these incidents combined could lead to chronic immune suppression and to the manifestation of disease. Molecular and bacteriological analysis confirmed the presence of opportunistic bacteria (e.g., *Aeromonas hydrophilia*) in the wounds of ill fish. 

## 5. Conclusions

Paradise fish with their complex behavioral repertoire [3,5,6,16,17,18,19,20,21,22,23,24,25,26] are a promising future model organism for behavioral genetics. However, in order to turn them into a modern model organism, it is essential to establish standardized protocols for housing and husbandry [61]. 

Here, we presented a summary of our efforts that led to a stable breeding population of paradise fish in the animal facility of ELTE Eötvös Loránd University. Our results demonstrated that appropriately adjusted water pH, feeding regime and stocking densities during larval rearing and adult housing can significantly improve the conditions and survival rate of this species under laboratory conditions. We are confident, therefore, that the improved housing conditions and husbandry routines outlined above meet the general fish welfare guidelines [27] and will be of great benefit to other groups who want to start experimenting with this exceptional species.

## Figures and Tables

**Table 1:** Summary of the water quality parameters and husbandry routine in the facility.

Water Quality Parameters and Husbandry Routine	
Air temperature	24.0–28.0 °C
pH	7.0–7.5
Conductivity	350–450 µS
NO_2_^−^	<0.06 mg/L
NO_3_^−^	<15–20 mg/L
NH_3_ (ammonia)	<0.05 mg/L
Fish feeding < 5 months	3× daily
Fish feeding > 5 months	2× daily
Tank cleaning larvae	<1 month—daily
Tank cleaning juvenile	>1 month—weekly
Tank cleaning >5 months (adults)	weekly (clean and siphon), with half water volume change
Adult fish holding density	10–12 fish/30 L
Light cycle	13 h Light:11 h Dark

**Table 2:** Survival of paradise fish larvae is dependent on stocking densities and feeding regime (survival shown as the percentage of the number of individuals reaching adulthood compared to the total number of larvae at the beginning of the respective rearing trial).

3 L Tanks, 10 Larvae/3 dL, *Artemia* from 3 wpf	3 L Tanks, 7 Larvae/3 dL, *Artemia* from 3 wpf	3 L Tanks, 5 Larvae/3 dL, *Artemia* from 6 dpf	3 L Tanks, 2–4 Larvae/3 dL, *Artemia* from 6 dpf	5 L Tanks, 1–1.5 Larvae/dL with Sponge-Water Filter	10 L Tanks, 1–2 Larvae/dL with Sponge-Water Filter
~1% (4/400)	~9% (31/350)	~28% (99/350)	~36% (65/180)	0% ^1^ (0/360)	0% ^1^ (0/450)

^1^ all the larvae died within 1–5 weeks post-fertilization (wpf). dpf = days post-fertilization.

**Table 3:** The reproductive behavior of *M. opercularis*, see Reference [5] for details.

Behavior	Male	Female
Nest-building + Courtship	Spread fins (DIS or LHT) Opercular erection (OPE) Brightening body color Territorial defense (BIT) Bubble nest build (A-B) Intense lateral spread display (LHH or LHT) Leads female to nest (LEN)	**Refusal**: Spread fins (DIS or LHT), Opercular erection (OPE), Swim away, hide (SWI or ESC) **Acceptance**: Pale body color, Oblique movements (OBM), Vertical waggle (VEW), Follow male
Spawning	Circle under nest (CIR) Anabantoid embrace (ANE) Ejaculation + Sinking (SIN) Release female	Circle under nest (CIR) Anabantoid embrace (ANE) Egg release + sinking (SIN)
Nest care/Guard	Collect eggs and put them into the nest (PIC) Blow more bubble (A-B) Chase away female (BIT) Guard nest (BIT) Clean and move eggs and embryos (PIC)	Either tries to help the male and is chased away (ESC) or Uninterested in nest care from start

For a complete list of behavioral elements and the corresponding abbreviations, see Table A1.

**Table 4:** A summary of the positive results in our health diagnostics.

Pathogen	Bacteriology	PCR
*Aeromonas hydrophila*	+	+
*Pseudomonas alcaligenes*	+	ND
*Aerococcus* spp.	+	ND
*Comamonas* spp.	+	ND
*Mycobacterium* spp.	ND	+
*Mycobacterium chelonae*	ND	+
*Mycobacterium fortuitum*	ND	+

“ND” = no data on specific pathogen (in specific test type); “+” = positive test for the specific pathogen.

## Data Availability

Not applicable.

## Supplementary Materials

The following are available online at https://www.mdpi.com/2076-2615/11/3/786/s1, Video S1: Paradise fish fight; Video S2: Paradise fish fight.

## Appendix A

**Table A1:** A list and short behavioral description (ethogram) of the most common behavioral elements in paradise fish.

Element	Abbreviation	Description	Source
Active elements			
Escape	ESC	Moving rapidly away from strong stimulus source.	[16]
Swim	SWI	Normal or fast locomotion with no special orientation.	[16]
Move	MOV	Slow, short-range locomotion with no special orientation.	[16]
Creeping	CRE	Slow, forward movement propelled only by the pectoral fin.	[16]
Staccato	STA	Quick starts and sudden stops, with no special orientation.	[16]
Leaping	LEP	Quick move, propelled by the caudal fin.	[16]
Erratic movement	ERA	Zig-zag locomotion performed on the bottom.	[16]
Jumping	JUP	Quick jump from a confined space, out of the water	[16]
Air gulping	A-G	Air gulping at the surface.	[16]
Air bubbling	A-B	Similar to air gulping but a few big bubbles are released through the gills or mouth. Most often associated with nest-building.	[16]
Approach	APR	Slow movement, oriented to a particular object.	[16]
Pick	PIC	Object picked with the jaw.	[16]
Back	BAC	Slow, backward locomotion.	[16]
Passive elements			
Floating	FLO	The fish is floating 1–2 cm under the surface.	[16]
Hanging in midwater	HIM	The fish is floating > 2 cm under the surface.	[16]
Resting	RES	Fish rests at the bottom, with pectoral fins fanning slowly.	[16]
Freezing	FRZ	Fish is motionless at the bottom.	[16]
Freezing under the surface	FUS	Fish is periodically motionless near the surface, and gulps air in-between.	[16]
Social elements			
Social orientation	ORI	Slow locomotion around a conspecific.	[16]
Oblique plan floating position ^1^	OBF	Submission posture, the body is 20–40 degrees inclined from the horizontal plane, only pectoral fins are fanning, other fins are closed.	[16]
Lateral head-head display ^2,3^	LHH	Fish are oriented in the same direction with one being slightly behind.	[18]
Lateral head-tail display ^2,3^	LHT	Fish (usually males) are oriented in opposite directions. Sometimes they also slowly start circling.	[18]
Lateral head-tail display with shaking ^2^	SHA	Similar to LHT but involves fast circling and descent to the bottom.	[18]
Frontal Display ^2^	FRD	Faces the opponent with unpaired fins extended. Body angle towards the opponent close to perpendicular.	[5,18]
Opercular Erection ^2^	OPE	Opercular erection, the opercular ocelli are visible. Usually performed by competing males during LHH, LHT or FRD.	[5,18]
Biting	BIT	The fish uses its jaws to inflict wounds by damaging the epidermis of the other fish	[5]
Charge	CHA	The fish swims fast in the direction of the opponent.	Described in the context of *B. splendens* [69]
Display at distance	DIS	Fish stay in head-tail position, with erected tailfin. Distance is larger than one body length.	[18]
Parallel swimming	PAS	The two fish swim closely to each other into the same direction.	[18]
Tail-beating	TAB	Occurs occasionally during LHH and LHT and consists of undulating thrusts of the tail towards the other fish	[5]
Mouth-lock	MOU	Occurs during intense fighting after FRD and OPE. Males lock their jaws and sink to the bottom of the tank. It can last from several seconds up to 2 min.	[18]
Lateral vibrating	VIB	Rapid undulatory movement that occurs when the pair is in head-to-head or head-to-tail position. Usually used by non-receptive females.	[5]
Circling	CIR	Male curves his body and slowly swims in a circle. Female follows with her snout at his dorsum.	[5]
Oblique plan movements	OBM	Median fins are commonly folded, the caudal fin often droops, and the fish often tilts laterally. Occasionally, the body may be tilted vertically, either upward or downward.	[5]
Vertical waggle	VEW	Low-frequency undulating movements of the caudal fin while the fish is in (near) vertical position. Often performed by receptive females.	[5]
Leading to the nest	LEN	After an LSD male swims slowly to the nest, with median fins erect.	[5]
Anabantoid embrace ^4^	ANE	Female moves into a U-shape flexture, with her snout protruding beyond the male’s dorsal fin. The male clasps the female, quivering of the body starts in both and they roll over. Genital pores move close to the bubble nest, gametes are released.	[5,7,11]
Swimming inhibition	SIN	After spawning, male releases the female and both become immobile, even start to sink.	[5]

^1^ In Reference [16], this is called OBQ, but we wanted to differentiate it from OBF; ^2^ In Reference [16], these are treated together as a single behavioral element, Display (DIP); ^3^ In Reference [5], these are treated together as a single behavioral element, Lateral Spread Display (LSD); ^4^ In Reference [5], this is called “Mounting, Clasping and Roll”.

## Appendix B

### Appendix B.1. Detailed Fin-Clipping Protocol

#### Appendix B.1.1. Required Reagents and Tools

- Lidocaine powder (Sigma L7757);
- Ethyl 3-aminobenzoate methane sulfonate/Tricaine powder (Sigma E10521);
- Ethanol, min. 70%;
- Fin-clipping board + blade + forceps;
- Anesthesia tank (anesthetic and analgesic);
- Recovery tank (analgesia and treatment tank).

#### Appendix B.1.2. Stock Solutions

- Lidocaine stock solution (500 mg/L) prepared fresh weekly with filtered water (or purified reverse osmosis (RO) water). Keep the stock solution in the dark or in a covered glass bottle at 4 °C.
- Tricaine (MS-222) stock solution (4 g/L final concentration) [70], containing 400 mg of MS-222 in 97.9 mL purified or reverse osmosis (RO) water: buffered to pH 7.5 with 2.1 mL of 1 M Tris (pH 9). Keep the stock solution at 4 °C for use in 1–2 weeks or at −20 °C for prolonged storage.

### Appendix B.2. Procedure

Prepare a 120 mg/L Tricaine (MS-222) and 4–5 mg/L lidocaine solution (diluted in fish water) and add them to the anesthesia tank. Prepare 4–5 mg/L lidocaine solution (diluted in fish water) in the recovery tank.

Place the fish into the anesthesia tank first. They will become immobile in approximately 4–5 min. Place the immobilized fish to the fin-clipping board and use the blade and forceps to cut off a small portion of the caudal fin for later analyses. It is important to start the fin-clipping process only when the fish is anaesthetized to stage 4–5 level of anesthesia (i.e., loss of equilibrium and no reaction to stimuli).

Return fish to the pre-prepared recovery tank. They will start moving within 1.5–2 min: first, they start to breathe air from the surface and start to swim, and after 4–5 min, they will get their equilibrium completely back as well.

When working with multiple individuals, always use 70% ethanol to sterilize the fin-clipping board, the blade and forceps between fish.

Fish can be kept in the recovery tank for up to 24 h. If longer storage is necessary, change the water in the recovery tank with the addition of a 2–2.5 mg/L concentration of lidocaine for another day before returning the fish back to its holding tank.
